# Proteomics: A New Research Frontier in Forensic Pathology

**DOI:** 10.3390/ijms241310735

**Published:** 2023-06-27

**Authors:** Matteo Antonio Sacco, Isabella Aquila

**Affiliations:** Department of Medical and Surgical Sciences, Institute of Legal Medicine, University “Magna Graecia” of Catanzaro, 88100 Catanzaro, Italy; matteoantoniosacco@gmail.com

Proteomics, the study of proteins and their functions, has revolutionized the field of forensic science by providing a powerful approach to identify and characterize proteins for various aims. By using proteomics, postmortem bodily fluids, tissues, and organs can be thoroughly and uniquely analyzed for protein identification and characterization. Exploring the application of proteomics in forensic pathology and autopsy analysis is the aim of this Special Issue. In addition, this Special Issue targets the following questions: how can proteomics be used in forensic autopsy analysis and pathology? How can forensic issues be resolved through protein characterization and identification? Finally, what advantages are there in using proteomics during forensic investigations?

Proteomics is now being utilized for forensic purposes as a promising field of study. According to the literature, proteomics has been widely applied in forensics to analyze and determine body fluid and tissue, estimate postmortem intervals, and establish individual identity [1]. Human samples, including blood, hair, bone, and fingernails, have been subject to proteomic analyses in recent years [1]. However, the application of forensic proteomics may run into obstacles, such as unidentified samples and contaminants [2]. Moreover, specific procedures are necessary when preparing a sample, which limits the generalized use of proteomic analysis in forensics. The advantage of proteomics lies in its ability to provide a more comprehensive overview of a sample than genomic analysis [2]. In addition, proteomics is an excellent option when nucleic acids are missing or deteriorated [2]. Thus, when DNA is unattainable, ambiguous, or non-existent, proteomics serves as a viable alternative forensic method [3]. Additionally, the literature describes other potential applications. For example, proteomic analyses of tissue residue from bullets can evaluate the organs that one particular bullet struck [2].

The use of proteomics in determining the cause of death can prove to be quite advantageous. For instance, with regard to drowning incidents, levels of apolipoprotein A1 (ApoA1) and alpha-1 antitrypsin were measured for ROC curve construction. ApoA1 levels were more predominant in the group who drowned, whereas alpha-1 antitrypsin levels showed the opposite trend. This suggests that analyzing protein biomarkers within blood samples could be useful in identifying the potential cause of death resulting from drowning [4]. Various protein markers have been examined in terms of postmortem diagnosis for different causes of death. Even though a single protein has not been able to effectively differentiate the cause of death with precision, specific levels of protein expression were studied and found to have applications in distinguishing between different causes of death [5]. Furthermore, proteomic biomarkers are useful for directly gauging the physiological conditions that existed at the time of death [4]. For instance, the reduction in the level 14-3-3 isoforms in the medullary serotonin system identified in cases of sudden infant death syndrome (SIDS) could potentially be used as a biomarker for SIDS risk in living infants [2,6,7].

The application of proteomics to estimate postmortem interval (PMI) is gaining traction. Through analyzing the postmortem changes that occurred, proteins within the tissue samples are identified and compared to a database that shows the rate at which they degrade over time. This technique shows promise in accurately determining the time of death for a crime scene investigation or for forensic purposes. By identifying specific proteins within a tissue sample, this allows for a more unique and reliable way to estimate PMI as opposed to traditional methods that may have a wider margin of error. Moreover, the spectrum of target proteins for the purpose of PMI estimation is still limited [6,7]. For instance, the identification of proteins, such as GAPDH and eEF1A2, that undergo predictable proteolysis in the early and mid-postmortem phase has opened up new avenues for the application of protein-based analysis for time since death estimation. SDS-PAGE and Western blot experiments conducted by researchers have verified the potential of GAPDH and eEF1A2 as future markers for postmortem alterations in skeletal muscle [6]. Also, other proteins such as alpha-enolase, malate dehydrogenase, and peroxiredoxin 2 have been correlated with postmortem interval (PMI) [2]. Also, various biological fluids and samples have been used. For instance, proteomic investigation of cerebrospinal fluid (CSF) may be used for postmortem interval estimation by comparing the proteomic profiles of CSF before and after death, while levels of serotonin and tryptophan hydroxylase can also be used to investigate SIDS [2].

In conclusion, proteomics holds enormous potential in forensic investigations, as it can provide additional information and help fill in gaps in cases where DNA profiling is not sufficient. Proteomics has already shown its worth in various investigations and has the potential to contribute significantly to forensic investigations [5]. However, empirical validation is necessary for making proteomics more accepted in forensics [5]. Fortunately, advancements in instrumentation and bioinformatics are improving forensic proteomics, so that its application could be a valid routine tool in the next few years.
